# Phylogeny Best Explains Latitudinal Patterns of Xylem Tissue Fractions for Woody Angiosperm Species Across China

**DOI:** 10.3389/fpls.2019.00556

**Published:** 2019-05-03

**Authors:** Jingming Zheng, Xia Zhao, Hugh Morris, Steven Jansen

**Affiliations:** ^1^Beijing Key Laboratory for Forest Resources and Ecosystem Processes, Beijing Forestry University, Beijing, China; ^2^Laboratory for Applied Wood Materials, Empa-Swiss Federal Laboratories for Materials Testing and Research, St. Gallen, Switzerland; ^3^Institute of Systematic Botany and Ecology, Ulm University, Ulm, Germany

**Keywords:** xylem space allocation, vessel, fiber, parenchyma, latitude, temperature

## Abstract

Investigating space allocation patterns of plant secondary xylem along a latitudinal gradient is useful to evaluate structure-function tradeoffs in woody angiosperm xylem. An anatomical dataset including 700 woody angiosperm species across China was compiled together with latitudinal and climate data for each species. The relative tissue fractions of vessels, fibers, and parenchyma (including ray and axial parenchyma) in xylem were analyzed to determine the effect of latitudinal differences and phylogeny on anatomical variation. The analyses revealed a trade-off between vessel and non-vessel fraction across latitude, with tissue fraction trade-offs mainly occurring between vessels and fibers, and between fibers and total parenchyma. Among 13 climate variables, thermal indices generally had greater explanatory power than moisture indices in bi-variate models for all cell types, while mean annual temperature, mean temperature of the coldest month, and annual actual evapotranspiration were included in the top multi-variate models explaining variance of different tissue fractions. Phylogeny and climate together explained 57–73% of the total variation in xylem space occupancy, with phylogeny alone accounting for over 50% of the variation. These results contribute to our knowledge of wood structure-function and are relevant to better understand forest response to climate change.

## Introduction

The structure of wood xylem is closely related to its functions. Angiosperm wood carries out three key functions simultaneously: water transport, mechanical support, and water and nutrient storage. These functions correspond largely to three principal cell types, namely vessels, fibers, and parenchyma. As the hydraulic function of stems is critical for plant survival and growth, xylem evolution is assumed to have proceeded toward increased efficiency and increased safety of water transport (Baas and Wheeler, 1996). Meanwhile, conflicting demands on xylem space allocation result in a range of trade-offs owing to adaptation in various ecological environments. For example, increased mechanical support often decreases water conduction efficiency but may contribute to embolism resistance (Hacke et al., 2001; Pratt and Jacobsen, 2016). Despite various recent studies on functional trade-offs in wood (e.g., Gleason et al., 2016b; Pratt and Jacobsen, 2016), xylem space allocation represents a rather new view on this topic that deserves due attention because xylem volume is limited and space must be shared by different cell types (Bittencourt et al., 2016; Brodersen, 2016; Gleason et al., 2016a). Although previous papers have confirmed the existence of trade-offs between tissue fractions in xylem (Zheng and Martínez-Cabrera, 2013; Ziemińska et al., 2015; Morris et al., 2016b), none of them attempted to explore trends of those trade-offs along a large latitudinal gradient and the role climate and phylogeny played in it as this paper aimes to do.

Woody angiosperms have evolved for more than 100 million years, during that geologic time span there have been many changes in climate and topography (Martínez-Cabrera et al., 2017). Evolution of multicellular vessels, for instance, has arisen independently in several clades (Carlquist, 2012). Despite convergent evolution in xylem specialization (Baas and Wheeler, 1996), determining the impact of phylogeny on xylem space allocation is important to unravel the potential influence of climate on xylem tissue fractions. A comparative study on patterns of six wood anatomical features indicated that wood anatomy supports molecular phylogenetic analyses using the Angiosperm Phylogeny Group (APG) system (Baas et al., 2000). However, most wood anatomical studies focussed exclusively on binary and categorical traits, such as the presence or absence of vestured intervessel pits, or the type of perforation plate, with little attention given to quantitative traits such as xylem tissue fractions. Moreover, parallel development of some xylem features is generally accepted, which implies that ecological adaptation to the environment (climate) plays a large role in the variance of xylem characteristics (Baas and Wheeler, 1996; Baas et al., 2000; Rita et al., 2015). For instance, the variation in wood structure among 325 tree species was found to be constrained by phylogeny, whereas the strategies of adjusting wood to enable fast growth rate were similar for unrelated species (Hietz et al., 2017). To what extent phylogeny plays a major role in explaining xylem space allocation variance along latitude gradients is largely unknown.

Macroclimatic variation along latitudinal gradients provides an excellent natural laboratory to investigate the role of climate (especially temperature and precipitation) and the potential effects of global warming on terrestrial organisms (Frenne et al., 2013). Latitudinal variation of many plant traits and their correlation with climate have been studied on large spatial scales, such as leaf size (Wright et al., 2017), plant height (Moles et al., 2009), and wood density (Chave et al., 2009). These studies generally supported shifts in traits with changing latitude, and found that temperature has a stronger influence on these traits compared to precipitation, implying that variation of plant traits along a latitudinal gradient is largely related to temperature rather than other climate variables (Frenne et al., 2013; Moles et al., 2014). There are a number of wood anatomical studies of species under different climates (Fichtler and Worbes, 2012; Ziemińska et al., 2013), and on latitudinal and altitudinal trends of vessel features (van der Graff and Baas, 1974; Baas et al., 1983; Noshiro and Baas, 2000, Liu and Noshiro, 2003; Wheeler et al., 2007). However, none of these have looked at the effects of climate on space allocation patterns or xylem tissue fractions (but see Morris et al., 2018).

China hosts a huge biodiversity, including many endemic species (McNeely et al., 1990). China also boasts the most diverse climate biomes, ranging from alpine tundra to tropical forests, and from humid forests to arid deserts (Wu, 1980). Moreover, since the wood anatomy of over a thousand of Chinese woody plant species has been published over the last decades, the anatomical information available provides an interesting opportunity to test hypotheses on large-scale patterns into xylem space allocation of woody plants under seasonal climate.

**FIGURE 1:**
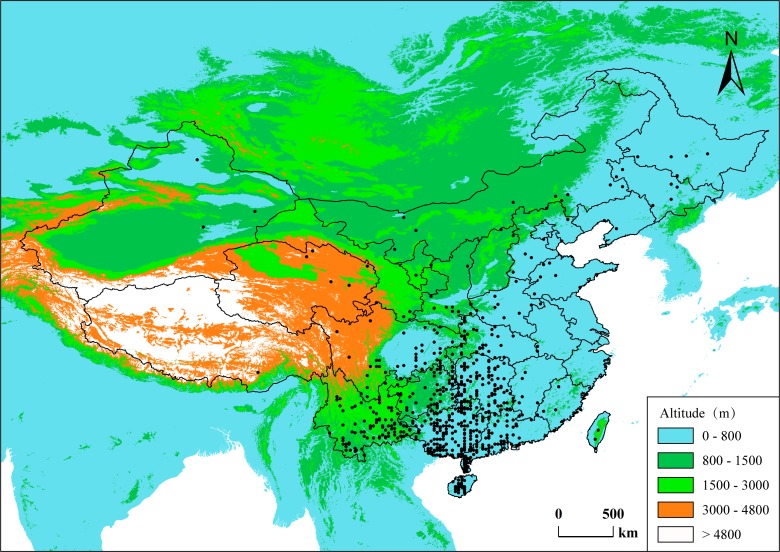
Spatial distribution of the data in this study. Each dot represents a species’ central distribution location on land of China, using the mid-latitude and mid-longitude values of the species’ range in the country as coordinates.

The primary goal of this study was to quantitatively explore latitudinal trends of xylem tissue fractions, testing the hypothesis that phylogeny and climate both contribute to the variance of xylem space use along a latitudinal gradient. We compiled a dataset of anatomical data from 700 woody angiosperms from across China (Figure 1). The dataset includes tissue area percent of vessels, fibers, and parenchyma as seen in transverse sections of stem wood, as well as mean vessel diameter and mean vessel density. Meanwhile, latitude and 13 climatic indices were matched for each species with the anatomical data. We predict that major differences in climate along a broad latitudinal range have an effect on the hydraulic demand and plant hydraulic architecture, which could be reflected in a trade-off between vessel tissue fraction and non-vessel xylem fraction. We also postulate that both phylogeny and climate contribute to the variance in tissue fractions owing to woody species’ being under ecological adaptation to climate on a large spatial scale.

## Materials and Methods

### Data Collection

We compiled wood data from 794 Chinese tree species (Yang et al., 2001, 2009). Most tree samples were taken from the Eastern Monsoonal climate zone in China, where the temperate, subtropical, and tropical natural forests are distributed (Wu, 1980). The anatomical data was mainly collected from publications by The Chinese Academy of Forestry, which hosts the largest wood collection in China, and the wood anatomical traits were measured following their methods. In brief, most angiosperm tree species were sampled from mature trees with a DBH over 20 cm in natural forests. The sampled disks were collected at a height of 1.3m. These disks were cut, from pith to bark, into six equal parts according to the equidistance method and anatomical samples were taken from the middle of the outermost part (the closest to the bark). The entire span of one growth ring was sectioned with a microtome. Wood anatomical traits were measured using a light microscope and analyzed with an image analysis system (Q570). For vessel diameter and vessel density, 100 vessels were measured in earlywood and latewood of each sample. Other important anatomical traits measured were the proportion of cross sectional area occupied by vessels, fibers (including tracheid), rays, and axial parenchyma. To measure tissue fractions of cross sections, images were taken with a Q570 image analyser. The percentage area of each cell type, however, was manually determined for 1 mm^2^ (Yang et al., 2001). All species names were verified using the Plant List^1^ to correct for synonyms, while synonyms were removed. In total we obtained wood anatomy data for 780 woody angiosperms species (Appendix 1). The anatomical data were deposited in TRY^2^ (Kattge et al., 2011).

As the sampling locations were not recorded for most of the species in the database, climate data for the natural distribution range in China were retrieved for each species. Among the 780 species in our database, climate information for 700 species over their range were extracted from the Atlas of Woody Plants in China (Fang et al., 2009). This book provided mean values for thirteen climatic variables across the range of each species on county level, as well as the species’ distribution map at the country level. The climate variables included three groups. (1) The thermal indices: mean annual temperature (MAT, °C), annual biotic temperature (ABT, °C), potential evapotranspiration (PET, mm), Kira’s warmth index (WI, °C ⋅ month) and coldness index (CI, °C ⋅ month), mean temperature of the warmest month (MTWM, °C), and mean temperature of the coldest month (MTCM, °C). (2) The humid/arid indices: mean annual precipitation (MAP, mm), precipitation in the warmest quarter of the year (PWQ, mm), and precipitation in the coldest quarter of the year (PCQ, mm). (3) Integrative indices included annual actual evapotranspiration (AET, mm) and moisture index (Im), and vegetation net primary production (NPP, g.a^-1^.M^-2^) estimated using the CASA model (for details, see Fang et al., 2009). In addition, maximum and minimum latitude and longitude for each species were also extracted from the distribution map to calculate the latitude and longitude midpoints of each species’ range (Appendix 1). Elevation could not be integrated in our analyses because we largely lack altitudinal gradients in our dataset. However, since most of the species of our dataset were distributed in the eastern part of China where altitudinal gradients are largely limited, it is reasonable to assume that altitudinal gradients play a less significant role than latitudinal gradients, especially under temperate climate in China. Only 19 species with a major distribution in western part of China were included in our dataset (e.g., *Quercus semecarpifolia, Rhododendron delavayi, Sorbus tianschanica, Populus euphratica*).

### Data Analysis

We build a phylogenetic tree for 780 species in this study using the R package “brranching” (Chamberlain, 2016). The stored super-tree was “R20120829” and the branch lengths of the phylo-tree were calculated using the BLADJ algorithm in phylocom (Webb et al., 2008). The tissue fractions (refereed as CFs afterward) were square root-transformed to increase normality in the data, before statistical analyses were performed with R.3.2 (R Core Team, 2015). Based on the phylo-tree obtained (Appendix 2), phylogenetic signals of quantitative traits were calculated as Pagel’s lambda using the “phylosignal” function in the R package “phytools” (Revell, 2012), phylogenetic independent contrasts for CFs were calculated using the AOT algorithm in phylocom (Webb et al., 2008), and the association between PICs for each cell type was analyzed using the “corr.test” function with the R package “psych” (Revelle, 2017).

To describe different aspects of xylem architecture, we calculated the *F* and *S* vessel metrics according to Zanne et al. (2010). *F*, which is the product of the mean vessel size (A, mm^2^) times the vessel number per unit area (N, mm^-2^), measures the wood fraction that is occupied by the vessel space, which is interpreted as directly measured vessel tissue fraction in this paper. Increases in *F* should be correlated with lower mechanical strength (Zanne et al., 2010). *S* is the ratio of the same anatomical traits (A/N; mm^4^), and measures the variation in vessel composition. A higher *S* reveals a greater contribution of large vessels to water conduction in a given area, and therefore indicates increased water transport efficiency, but brings a heightened risk of frost-induced embolism. These two metrics represent orthogonal axes of variation (Zanne et al., 2010). All indices were log_10_-tranformed to increase their normality for statistical analyses.

We conducted two types of phylogenetic analyses to account for the possible influence of phylogeny on the results that may explain the relationship between CFs and climatic factors. Firstly, the phylogenetic generalized least square method (PGLS) was used to build bi-variate and multi-variate models in a phylogenetic context, with the “gls” function in the R package “nlme” (Pinheiro et al., 2016), in which each tissue fraction was expressed as a function of climatic variables. In this method, the influence of phylogeny on a trait was included in the models’ correlation structure as a simple Brownian motion, thus various climatic variables for a trait could be compared within the same correlation structure. As there were 13 variables as potential predictors of each CF, we needed to reduce the number of predictors to minimize collinearity. We did a three-step procedure to estimate the combined effect of climate and traits on tissue fractions (Zheng et al., 2017). As most of the climatic variables are closely inter-correlated (Appendix 3), we started by reducing the initial pool of 13 climatic variables to a smaller number based on the results of the bivariate analysis. Firstly, the variables with an *R*^2^ < 0.02 in explaining CFs were excluded (see Appendix 4). Next, within each of three groups of climate indices, we used an AIC-based backward elimination to select the variables that best explained CFs variation. Lastly, all climatic predictors that survived the first two steps were included to build the full models, and AIC was used to select the most parsimonious model. As there are too many possible interactions that would overwhelm our available degrees of freedom in the full models, we did not include any interactions. Determination coefficients (*R*_phy_^2^) for each model were calculated using the maximum likelihood (ML) estimation. For our phylogenetic analysis, *R*_phy_^2^ was defined as the proportion of variation explained by the linear model, taking the correlation among observations into account, divided by that of the null model (intercept-only) with the same correlation structure in PGLS. Therefore, *R*_phy_^2^ can be regarded as a pseudo-R^2^.

For the second phylogenetic test, we used a mixed-model analysis of covariance (MACO) to partition contributions from phylogeny and climate (He et al., 2009). In contrast to the commonly used method of phylogenetically independent contrasts or phylogenetically general least square, this method has the advantage that the effect of phylogeny on CFs can be quantified as the percentage of variations explained by ancient lineages formed at different times in the evolutionary history. Using this method, we created our phylogenetic groups by “cutting” the tree at a given divergence time, since taxonomic groupings may adequately represent the pattern of evolutionary relationships between species, but not represent the rate of evolution. We created two contrasts, a first-order grouping cut at 59 million years before present (*n* = 85 groups, Appendix 2), and a second-order grouping cut at 28 million years before present (*n* = 292 groups). We chose these ages because they resulted in roughly the same number of groups, and thus degrees of freedom, as the families and genera used in the taxonomic analysis. In brief, this method was exactly analogous to the decomposition of the sums of squares in an analysis of variance (ANOVA), and the total sums of squares was partitioned into component sums of products for the above relationships. F-ratios of mean products ( = sum of products/degree of freedom) were used to test for significance. Using an ordinary rather than REML mixed-model analysis of covariance, we could obtain the sum of products that were additive, allowing us to express the influence of phylogeny and climate on CFs by the percent of the total sum of the products they explained (for details, see He et al., 2009). Consequently, the relative effects of phylogenetic and climatic factors on CFs could be compared (He et al., 2009).

## Results

### Descriptive Statistics of Xylem Tissue Fractions

**FIGURE 2:**
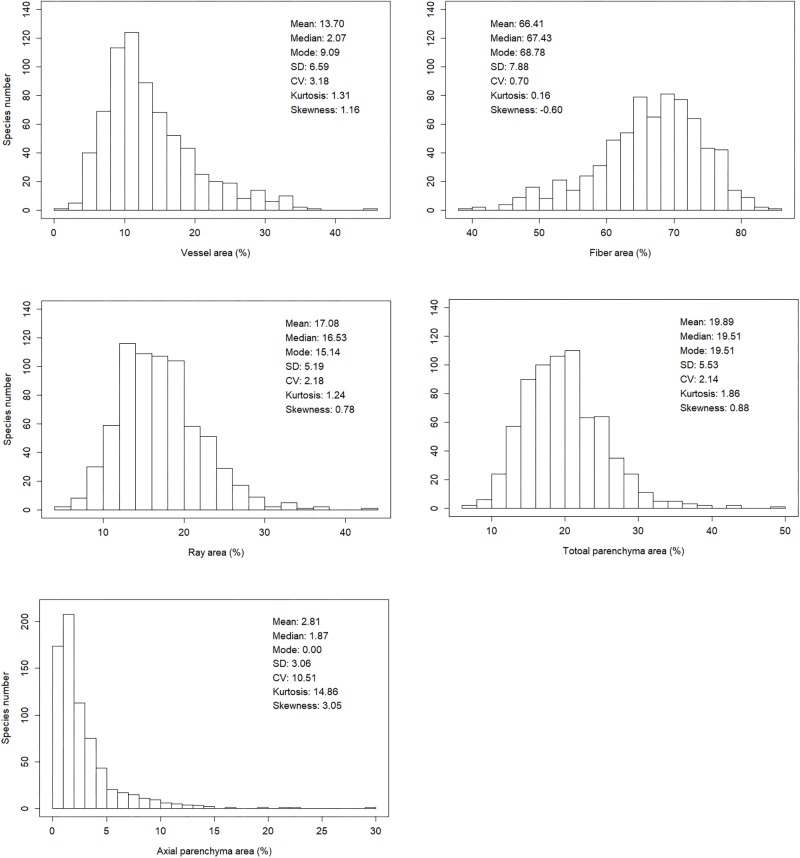
Frequency of observations for vessel fraction, fiber fraction, ray fraction, axial parenchyma fraction and total parenchyma fraction (i.e., sum of ray and axial parenchyma fraction).

Xylem tissue fractions varied greatly among the 700 angiosperm woody species. For vessel fraction, values range from 8.19 to 66.50%; for fiber fraction, values range from 63.08 to 92.74%; for ray parenchyma fraction, values range from 22.87 to 65.17%; for axial parenchyma fraction, values range from 0 to 54.31%; for total parenchyma fraction (sum of ray parenchyma and axial parenchyma fraction), values range from 26.23 to 70.34%. Frequency of values for axial parenchyma fraction is strongly right skewed while other four fractions were less skewed (Figure 2).

### Effects of Phylogeny on Xylem Tissue Fractions

All five xylem tissue fractions, i.e., vessel, fiber, ray, axial parenchyma, and total parenchyma (ray and axial parenchyma combined), showed significant phylogenetic signals (*P* < 0.001). Among the five tissue fractions, axial parenchyma had the highest value of lambda (0.895) followed by vessels (0.852), total parenchyma (0.760), fibers (0.732), and ray parenchyma (0.673).

### Latitudinal Pattern of Xylem Tissue Fractions

**FIGURE 3:**
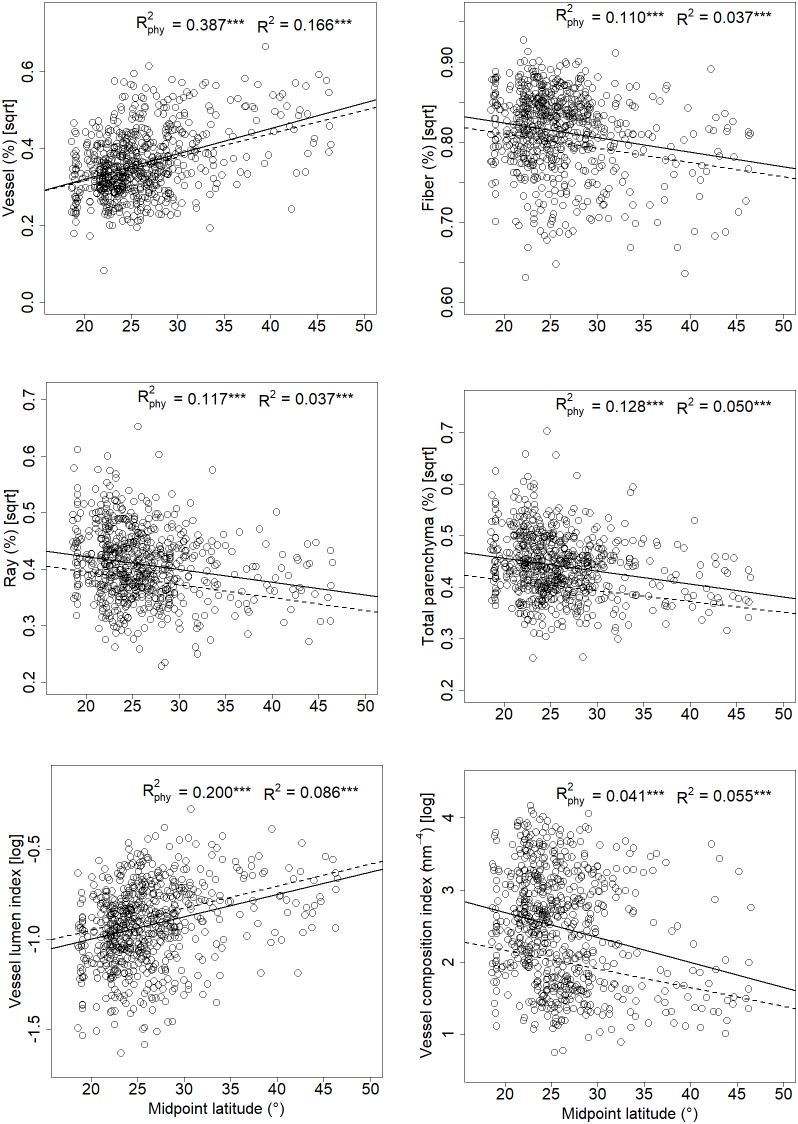
Relationships between xylem tissue fractions, *F, S*, and midpoint latitude for 700 wood angiosperm species in China. Each dot represents a single species. Dashed lines and solid lines represented linear regressions with and without taking phylogeny into account, respectively. “^∗∗∗^” indicates *p* < 0.001.

Tissue fractions of vessels, fibers, ray, and total parenchyma showed significant latitudinal patterns with and without taking phylogeny into account (Figure 3); and the AICs were lower for PGLS models than corresponding linear models (Appendix 4). Vessel fraction increased (0.67%∼44.22%) with rising latitude (N18.6°∼N72.1°), while tissue fractions of fibers, ray, and total parenchyma were higher at lower latitudes. No significant linear relationship was detected between axial parenchyma fraction and latitude (*P* = 0.718 and 0.053 for the phylogenetic and linear model, respectively). The other two variables describing vessel composition (*S*) and vessel lumen area (*F*) showed opposite latitudinal pattern, with *S* decreasing while *F* increasing with a rising latitude (Figure 3).

### Associations Between Xylem Tissue Fractions and Climatic Factors

**FIGURE 4:**
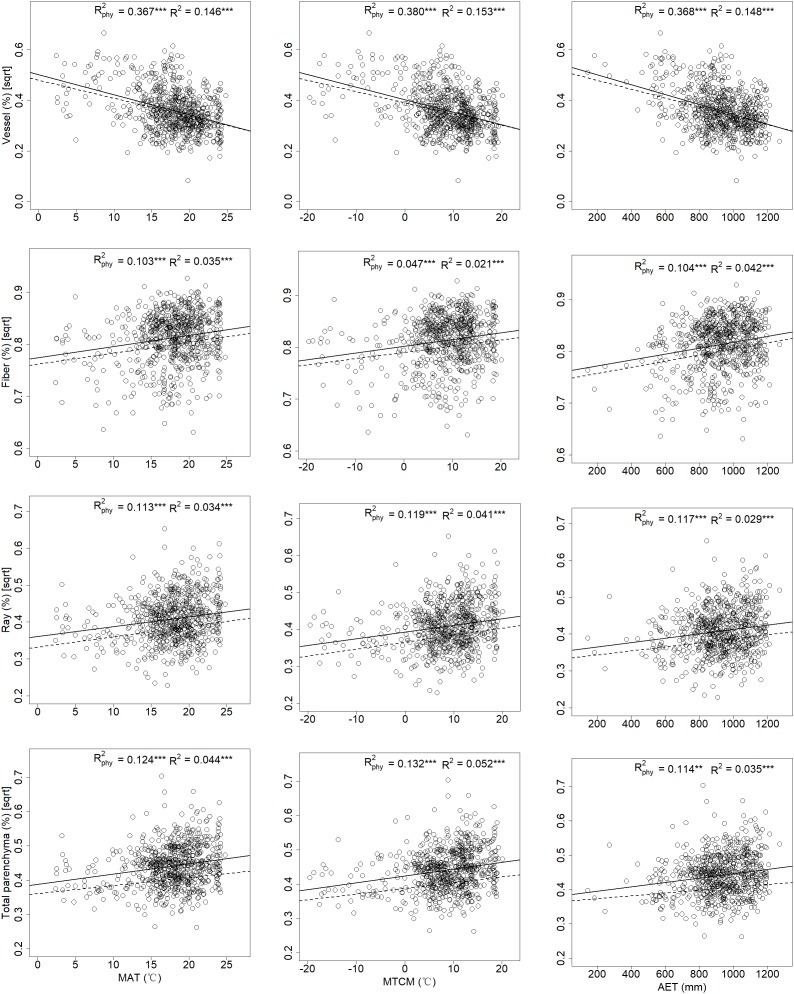
Associations of mean annual temperature, mean temperature of the coldest month, and annual actual evapotranspiration with xylem tissue fractions for 700 angiosperm species from China. Each dot represents a single species. Dashed lines and solid lines represented linear regressions with and without taking phylogeny into account. “^∗∗^” and “^∗∗∗^” indicates *p* < 0.01 and *p* < 0.001, respectively.

In the bi-variate models for tissue fractions and climatic variables, the majority of the 13 climatic indices correlated positively with vessels but negatively with fibers, ray, and total parenchyma fractions; however, the correlation between axial parenchyma fractions and any climatic index was not significant (Figure 4 and Appendix 4). MAT (mean annual temperature) and MTCM (mean temperature of the coldest month) had higher *R*_phy_^2^ than other temperature indices. AET (annual actual evapotranspiration) was the most influential index in integrative climatic indices, while three precipitation indices had less explanatory power compared to temperature indices for all tissue fractions in term of *R*_phy_^2^ (Appendix 4).

Among the top multivariate PGLS models with different tissue fractions as a function of climatic variables, MAT, MTCM, and AET were the only three climatic variables entered, while precipitation indices were excluded (Table 1). MAT was included in the top multivariate models for vessels, fibers, ray, and total parenchyma fractions, but not for axial parenchyma. AET was included in the models for both vessel and fiber tissue fractions, and MTCM for vessel, ray parenchyma, and total parenchyma tissue fractions. Climatic factors explained a larger proportion of vessel fraction variances (*R*_phy_^2^ = 0.401) than those of other tissue fractions.

**Table 1:** Best multivariate models for tissue fractions as a function of climatic predictors based on 700 woody angiosperm species from China.

	F-value	P	F-value	P	F-value	P	F-value	P
				
	VF		FF		RF		RAF	
MAT	91.95	<1e-04	23.47	<1e-04	24.91	<1e-04	18.19	<1e-04
MTCM	4.92	0.027	–	–	8.98	0.003	11.71	7e-04
AET	9.30	0.002	9.63	0.002	–		–	–
*R*_phy_^2^	0.40		0.10		0.12		0.13	
df	696		697		697		697	


*VF, vessel fraction; FF, fiber fraction; RF, ray parenchyma fraction; AF, axial parenchyma fraction; RAF, total parenchyma fraction; MAT, mean annual temperature; MTCM, mean temperature of the coldest month; AET, actual evapotranspiration.*

### Partitioning the Effect of Phylogeny and Climate on Tissue Fractions

**FIGURE 5:**
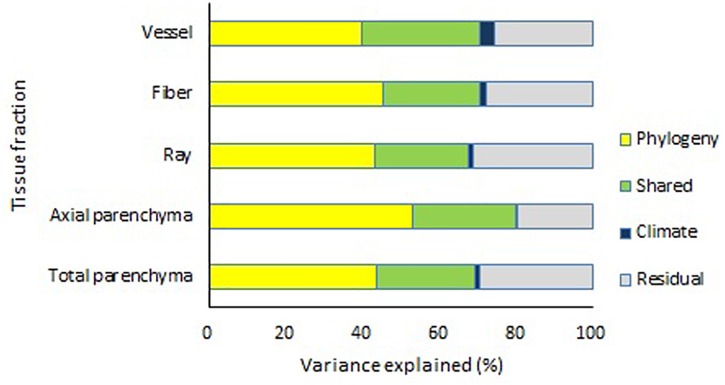
Effects of climate and phylogeny on vessel fraction, fiber fraction, ray fraction, axial parenchyma fraction, and total parenchyma fraction, expressed as percentage of sum of products explained.

Decomposition of covariance showed that phylogeny and climate together could explain about 59.7–72.8% of the variation for different tissue fractions (Figure 5). However, the explanatory power of climatic parameters only was very small, while phylogeny could explain about 50.8–72.2% variance of the tissue fractions (Appendix 5). For vessel fraction, phylogeny alone accounted for 50.8% of the variance, with climate alone accounting for 5.3% of the variance, and phylogeny and climate sharing 11.1% of the variance.

## Discussion

### Latitudinal Pattern of Xylem Tissue Fractions

Opposing trends between vessel and non-vessel tissue fractions along latitudinal gradients were found among angiosperm species across China, which supports our hypothesis. These spatial pattern of xylem tissue fractions also suggested that xylem space allocation shows at least partly adaptation of the plant hydraulic system in woody plants. Therefore, the trade-off between non-vessel and vessel tissue fractions reflects the hydraulic properties of the vessel tissue and the additional xylem tasks such as hydraulic capacity (Jupa et al., 2016), storage of non-structural carbohydrates by parenchyma (and living fibers) (Plavcová et al., 2016), and protection against pathogens (Morris et al., 2016a). On one hand, trees at high latitudes generally possess small vessel diameter to reduce the risk of frost-induced hydraulic failure (Zanne et al., 2014). Increased vessel density, however, and relatively high vessel tissue fractions could mediate reduction of water conductivity for trees at high latitudes. Since hydraulic conductivity scales to the fourth power of the vessel diameter, but linearly with vessel density according to the Hagen–Poiseuille law, it is possible that increased vessel density alone is not enough to compensate the effect of narrow vessel diameters. Moreover, vessel diameter is well known to be negatively correlated with vessel density. On the other hand, reduced fiber and ray fractions in trees located at higher latitudes might be associated with reduced growth rate or plant size compared to trees in tropical regions. However, changes in fiber and ray tissue fractions would not imperil xylem’s multiple functions that are essential for a plant’s life history under various climatic conditions, as diversified xylem space allocation in angiosperms could be viewed as a strategy to adapt to different environments (Bittencourt et al., 2016). For instance, wide variance of tissue fractions occurred in a rather narrow range of wood density for 69 Australian tree species (Zieminska et al., 2015). No clear latitudinal pattern of axial parenchyma was found, which is probably due to a lack of tropical tree species in our data. Levels of axial parenchyma in tropical trees are generally higher than those of temperate trees, and on a global scale axial parenchyma fraction declines along latitude (Morris et al., 2016b).

### Trade-Offs Between Xylem Tissues andHydraulic Functions

The rising vessel fraction toward higher latitudes came to a great extent at the cost of decreased fiber fractions, which was associated with a change in total parenchyma fractions. PIC correlation analysis indicated that a strong association existed between vessel fraction and fiber fraction (*r*_pic_ = -0.70), and between fiber fraction and total parenchyma fraction (*r*_pic_ = -0.63), and only weak association between total parenchyma fraction and vessel fraction (*r*_pic_ = -0.08) (Table 2). Therefore, a major trade-off between vessel and fiber fraction occurred along a latitudinal gradient. This is in agreement with previous studies, which also showed that strong trade-offs existed between vessel and fiber fractions within the xylem space (Fichtler and Worbes, 2012; Zheng and Martínez-Cabrera, 2013; Ziemińska et al., 2013). It has been suggested that structural trade-off between parenchyma and fiber fraction is due to the relatively high proportion of both tissues in wood (Ziemińska et al., 2015). Our study agrees with the idea that a strong trade-off exists between parenchyma and fiber fractions. Taken together, these results suggested that a changing environment along a latitudinal gradient in China has a significant effect on the structural trade-offs between the three main cell types (vessel, fiber and parenchyma). Moreover, the structural trade-offs are directly linked to functional trade-offs, although each cell type may fulfill multiple functions rather than a specific function.

**Table 2:** Correlation coefficients among the five xylem tissue fractions studied based on 700 woody angiosperm species from China.

	VF	FF	RF	AF	RAF
VF		*-****0.72***	*-0.06*	*-****0.18***	*-****0.16***
FF	*-****0.70***		*-****0.52***	*-0.08*	*-****0.55***
RF	*-0.10*	*-****0.52***		*-****0.19***	***0.84***
AF	*0.08*	*-****0.30***	*-****0.13***		***0.34***
RAF	*-0.08*	*-****0.63***	***0.87***	***0.33***	


*Species-wise correlations are shown above and phylogenetic independent contrast (PIC) correlations below the diagonal. Significant level: italicized, <0.05; italicized and bold, <0.001. VF, vessel fraction; FF, fiber fraction; RF, ray parenchyma fraction; AF, axial parenchyma fraction; RAF, total parenchyma fraction.*

To further explore the changes in vessel composition along a latitudinal gradient, we analyzed the trends of the vessel composition index (*S*, mm^4^) and vessel lumen area index (*F*, unitless) along a latitudinal gradient. Zanne et al. (2010) showed that *S* could explain a large proportion of structural variance within the hydraulic system, and that variation in conductivity is more strongly influenced by *S* than by *F* (Zanne et al., 2010). Results showed that *S* decreased, whereas *F* increased significantly with rising latitude (Figure 3). The latitudinal trend of *F* is consistent with that of vessel tissue fraction, and both metrics have the same meaning, although *F* is calculated by multiplying vessel diameter measurements with vessel density data, while vessel fraction is measured directly. The higher value of *S* at low latitudes indicates that the conducting area is comprised of a few large vessels, which are highly efficient for long distance water transport, while low values of *S* at high latitudes indicate that the conducting area is comprised of many small vessels, which are resistant to embolism risk under freeze-thaw conditions (Zanne et al., 2014). Therefore, the opposite latitudinal trends of *S* and *F* suggest a trade-off between vessel size and vessel number in the hydraulic system of woody angiosperm species across latitude.

### Effects of Climate on Xylem Tissue Fractions

Climate could have an effect on xylem space use as most climatic indices were significantly associated with vessel, fiber, ray, and total parenchyma fractions, except for axial parenchyma fraction (Appendix 4). Generally, thermal indices have better explanatory power than moisture indices. For instance, in two bi-variate models explaining variance in vessel tissue fraction by mean annual temperature (MAT) or by mean annual precipitation (MAP), *R*^2^_phy_ of the former model (0.367) was higher than that of latter (0.301). A global study on parenchyma patterns found that MAT had a stronger influence on total parenchyma fractions than MAP, but no significant relationship was found between axial parenchyma and these two climatic indices (Morris et al., 2016b), which is in line with our results. We found that climatic variables also influence vessel and fiber fraction (Appendix 4). Moreover, the moisture index (Im), an indicator for regional aridity, was only weakly associated with vessel fraction (*R*^2^_phy_ = 0.058), showing that an aridity gradient is not obvious across the latitudinal range in China. Most of the woody angiosperms species in this study were distributed in the eastern part of China, where a monsoonal climate dominates with a synchrony of thermal and moisture conditions. This is consistent with the fact that water was generally not a limiting factor for trees, even in regions that experience freezing events (Bjorklund et al., 2017).

Temperature had an important influence on trade-offs between xylem tissue fractions along altitudinal gradients. The top multivariate models, which include MAT, MTCM and AET, support this point (Table 1). Temperature was a critical factor in limiting the production and differentiation of xylem cells in cold climates (Rossi et al., 2012). For instance, mean annual temperature and coldest temperatures were correlated with maximum plant height globally (*R*^2^ = 0.170∼0.195) (Moles et al., 2009). Our results showed that a correlation between MCTM and vessel and parenchyma fractions was higher than those between MAT and vessel and parenchyma fractions in relevant bi-variate models (Appendix 4), suggesting that winter temperature may influence xylem development, and thus xylem structure and functions. According to the growth limitation hypothesis (Körner, 1998), there is a minimum temperature that permits sufficient production of new cells and differentiation of xylem tissue in higher plants. An average daily temperature of 6 to 8°C is commonly considered to be a thermal boundary layer below which metabolic activities are inhibited (Rossi et al., 2012). In our dataset, c. 300 species had MTCM values lower than 8°C, thus explaining the close association between MTCM and vessel fraction as well as fiber, ray and total parenchyma fractions, which is in agreement with the growth limiting hypothesis. Furthermore, recent studies on wood formation showed that temperature could regulate the timing of cambial reactivation and xylem differentiation for temperate trees, thus temperature could regulate the important xylem and phloem functions of transport, storage, and mechanical support (reviewed by Begum et al., 2018). In addition, a decrease in temperature might be the critical factor in the control of latewood formation and the cessation of cambial activity (Begum et al., 2018).

In this paper, xylem tissue fraction demonstrates a clear pattern along a large latitudinal gradient, where phylogeny alone could explain approximately half of all variance in the traits studied. Our results thus suggest that xylem anatomy is conservative, being largely determined by evolutionary history rather than ecological conditions. We found it unexpected that climate alone has a respectively low explanatory power over tissue fraction variance given that bi-variated models indicated a significant correlation between climatic indices and tissue fractions in general. Commonly, climate had a strong influence on plant performance (such as size, phenology, productivity, and some hydraulic traits) on a global scale. For instance, theoretical hydraulic conductivity (*K*_th_) of 28 *Eucalyptus* species across Australia was significantly associated with aridity index (Pfautsch et al., 2016), while xylem vulnerability to embolism of *Fagus sylvatica* across Europe was associated with mean annual temperature and aridity (Schuldt et al., 2016). One possible reason for the low explanatory power of climatic indices on xylem tissue fractions could be that the climatic indices used in this paper represented a species’ average climatic condition over ones range rather than actual sample sites, while trees’ performance (including hydraulic function and xylem structure) might differ between their geographical center and margin (Stojnic et al., 2018).

Another possible reason for the low explanatory power of climate on xylem tissue fractions might lie in the modularity of angiosperms. Higher plant species grow through repeated increases in their modules (e.g., leaves, branches), so the effect of climate on the functions at the individual component level is likely compromised by the tree’s overall architecture. In commenting on why only a weak trade-off between xylem safety and efficiency was detected at the branch level in a global study by Gleason et al. (2016b), Brodersen (2016) suggested that hydraulic variables of stems and branches might not be enough to represent the whole tree’s hydraulic functioning, and integration of hydraulic data from along the entire hydraulic pathway would be useful. The same concept of modularity has also been described under different terms, such as hydraulic vulnerability segmentation (Johnson et al., 2016), hydraulic sectoriality (Zanne et al., 2006), and hydraulic redundancy (Ewers et al., 2007). Future studies should integrate structural anatomy of xylem and its multiple functions at different plant levels, such as at the cellular level (i.e., the role of pit membranes in water transport; Jansen et al., 2018), the tissue level (i.e., connection between different cell types, especially parenchyma and vessels), the branch level (i.e., autonomy of branch functions), and at the whole-plant level (i.e., the xylem continuum from minor roots to minor leaf veins).

## Conclusion

In this paper, we tested whether xylem tissue fractions show a trade-off along a latitudinal gradient in China, and how much of the variance in tissue fraction could be explained by phylogeny and climate. We found that vessel tissue fraction increased with rising latitude, while non-vessel fractions decreased, demonstrating a trade-off between vessel fraction and non-vessel fraction. Climate and phylogeny together shaped xylem space allocation, with phylogeny accounting for about half of the variance of vessel, fiber, ray and total parenchyma fractions while temperature, most notably freezing temperatures, had a considerable impact on vessel and total parenchyma fractions at higher latitudes.

## Author Contributions

JZ planned the study. XZ and JZ collected the data and did the statistical analyses. JZ, HM, and SJ wrote the manuscript.

## Conflict of Interest Statement

The authors declare that the research was conducted in the absence of any commercial or financial relationships that could be construed as a potential conflict of interest.

## Footnotes

1 http://www.theplantlist.org/

2 http://www.try-db.org
