# Supplementary material for: Phylogeny Best Explains Latitudinal Patterns of Xylem Tissue Fractions for Woody Angiosperm Species Across China
Source: Front Plant Sci. 2019 May 3;10:556. doi: 10.3389/fpls.2019.00556 (PMC6509232; doi:10.3389/fpls.2019.00556)
Supplement: Supplementary file 1 [file Table_1.DOCX]

Supplementary Material

Phylogeny best explain latitudinal patterns of xylem tissue fractions for woody angiosperm species across China

**Jingming Zheng^1*^, Xia Zhao^1^, Hugh Morris^2^, Steven Jansen^3^**

*** Correspondence:** Jingming Zheng: zhengjm@bjfu.edu.cn

**Appendices legend**

**Appendix 1.** Traits and environmental factors examined in this study, with reference to their acronyms, units, and definition.

**Appendix 2.** Phylogenetic-tree of the 700 woody angiosperm species studied based on stored super-tree “R20120829” in phylomatic program (http://phylodiversity.net/phylomatic/pmws). The green and the red line indicate the cutting age for 1st-order grouping and 2nd-order grouping, respectively.

**Appendix 3.** Pearson correlation coefficients among climatic variables. Blue color indicates negative correlation while red indicates positive, the colored area represent value of correlation coefficients, n=700 species. For a list of the acronyms, see Appendix 1.

**Appendix 4.** Summary of univariate models for explaining cell percentage with other traits and environmental factors. Variables in bold italic fond were those with p>0.05 or R_phy_^2^<0.02. In the R_2_ column, “-” denotes negative relationships, while others were positive ones. N=700 species.

**Appendix 5**

Summary of decomposition of covariance in a general linear model, using sums of products, for the effects of climate (MAT, MTCM and AET) and phylogenetic variation (1st-order grouping and 2nd-order grouping) on the five xylem tissue fractions (VF, BF,RF, AF and RAF). Explanatory terms are listed in the order of their entry into the model. Df: degree of freedom, %SP: percentage of total sum of products, Sig.: significance, ***: P ≤ 0.001, **: P ≤ 0.01, *: P ≤ 0.05, NS: P > 0.05.

**Appendix 1.** Traits and environmental factors examined in this study, with reference to their acronyms, units, and definition.

|  | Units | Description |
| --- | --- | --- |
| **Geography gradient** |  |  |
| Maximum longitude(MALO) | ° | Maximum longitude of a species’ distribution area |
| Minimum longitude(MILO) | ° | Minimum longitude of a species’ distribution area |
| Midpoint longitude(MPLO) | ° | Midpoint longitude of a species’ distribution area |
| Maximum latitude(MALA) | ° | Maximum latitude of a species’ distribution area |
| Minimum latitude(MILA) | ° | Minimum latitude of a species’ distribution area |
| Midpoint latitude(MPLA) | ° | Midpoint latitude of a species’ distribution area |
| **Traits** |  |  |
| Vessel fraction (VF) | % | Percentage of vessel area in a cross-section of wood |
| Fiber fraction (FF) | % | Percentage of fiber area in a cross-section of wood, including tracheids |
| Ray fraction (RF) | % | Percentage of ray area in a cross-section of wood |
| Axial parenchyma fraction (AF) | % | Percentage of axial parenchyma area in a cross-section of wood |
| Total parenchyma fraction (RAF) | % | Percentage of total parenchyma area in a cross-section of wood, including axial parenchyma and ray |
| Maximum height (H_max_) | m | Maximum height of a tree species |
| Vessel diameter (D) | mm | Mean tangential diameter of individual vessels |
| Vessel area (A) | mm^2^ | Mean vessel area, calculated as 3.1416*(D/2)^2^ |
| Vessel density (N) | mm^-2^ | Mean number of vessels in a transverse wood section |
| Vessel lumen fraction (F) | mm^2^.mm^-2^ | Sum the total conduit area, calculated as A*N |
| Vessel composition metric (S) | mm^4^ | The variation in vessel size, calculated as A/N |
| **Thermal indices** |  |  |
| MAT | ℃ | Mean annual temperature |
| ABT | ℃ | Annual bio-temperature |
| PET | mm | Potential evapotranspiration |
| MTWM | ℃ | Mean temperature of the warmest month |
| MTCM | ℃ | Mean temperature of the coldest month |
| WI | ℃·month | Warmth index, mean monthly temperature for all months that exceed 5°C |
| CI | ℃·month | Coldness index, mean monthly temperature for all months lower than 0°C |
| **Humid/arid indices** |  |  |
| MAP | mm | Mean annual precipitation |
| PWQ | mm | Precipitation of warmest quarter |
| PCQ | mm | Precipitation of coldest quarter |
| **Integrative climatic indices** |  |  |
| AET | mm | Annual actual evapotranspiration |
| NPP | g.a^-1^.m^-2^ | Vegetation net primary productivity, calculated using the CASA model over each species’ range |
| Im | / | Moisture index, similar to MAP/PET |

**Appendix 2.** Phylogenetic-tree of the 700 woody angiosperm species studied based on stored super-tree “R20120829” in phylomatic program (http://phylodiversity.net/phylomatic/pmws). The green and the red line indicate the cutting age for 1st-order grouping and 2nd-order grouping, respectively.


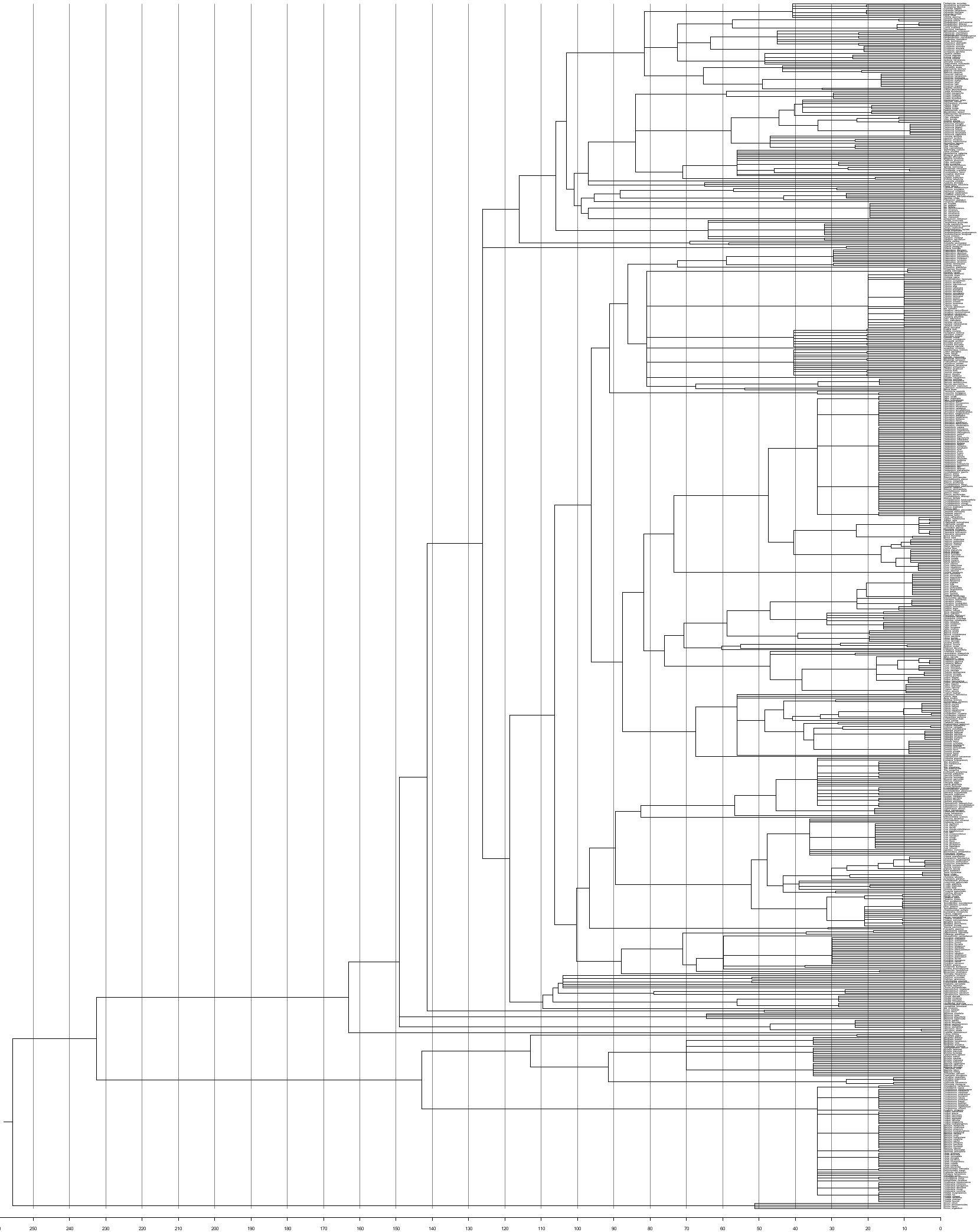


**Appendix 3.** Pearson correlation coefficients among climatic variables. Blue color indicates negative correlation while red indicates positive, the colored area represent value of correlation coefficients, n=700 species. For a list of the acronyms, see Appendix 1.


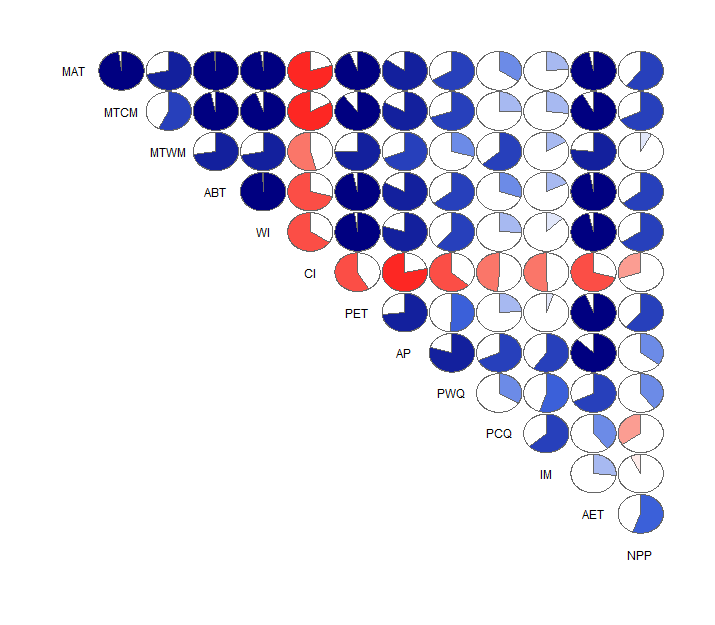


**Appendix 4.** Summary of univariate models for explaining cell percentage with other traits and environmental factors. Variables in bold italic fond were those with p>0.05 or R_phy_^2^<0.02. In the R^2^ column, “-” denotes negative relationships, while others were positive ones. N=700 species.

4.1 Vessel fraction

| Predictor | Phylogenetic model | | | | Non-phylogenetic model | | |
| --- | --- | --- | --- | --- | --- | --- | --- |
|  | p-value | R_phy_^2^ | lambda | AIC | p-value | R^2^ | AIC |
| **Geography gradient** |  |  |  |  |  |  |  |
| Maximum longitude(MALO) | <0.0001 | 0.145 | 0.820 | -1660.263 | <0.0001 | 0.051 | -1497.245 |
| Minimum longitude(MILO) | 0.003 | -0.033 | 0.850 | -1642.637 | <0.001 | -0.0169 | -1472.71 |
| Midpoint longitude(MPLO) | ***0.151*** |  |  |  | 0.05741 |  |  |
| Maximum latitude(MALA) | <0.0001 | 0.382 | 0.751 | -1754.394 | <0.0001 | 0.175 | -1595.348 |
| Minimum latitude(MILA) | <0.0001 | 0.215 | 0.790 | -1658.628 | <0.0001 | 0.083 | -1521.343 |
| Midpoint latitude(MPLA) | <0.0001 | 0.387 | 0.735 | -1734.298 | <0.0001 | 0.166 | -1588.044 |
|  |  |  |  |  |  |  |  |
| **Climate** |  |  |  |  |  |  |  |
| Thermal index |  |  |  |  |  |  |  |
| MAT | <0.0001 | -0.367 | 0.735 | -1711.768 | <0.0001 | -0.146 | -1571.194 |
| MTCM | <0.0001 | -0.380 | 0.729 | -1716.59 | <0.0001 | -0.153 | -1576.783 |
| MTWM | <0.0001 | -0.114 | 0.826 | -1648.897 | <0.0001 | -0.050 | -1496.616 |
| ABT | <0.0001 | -0.361 | 0.739 | -1711.864 | <0.0001 | -0.142 | -1567.997 |
| PET | <0.0001 | -0.313 | 0.759 | -1695.228 | <0.0001 | -0.118 | -1548.669 |
| WI | <0.0001 | -0.347 | 0.745 | -1706.811 | <0.0001 | -0.134 | -1561.445 |
| CI | <0.0001 | 0.252 | 0.781 | -1674.949 | <0.0001 | 0.107 | -1540.12 |
| Humid/arid index |  |  |  |  |  |  |  |
| MAP | <0.0001 | -0.301 | 0.765 | -1693.609 | <0.0001 | -0.120 | -1550.219 |
| PCQ | <0.05 | ***-0.019*** | 0.852 | -1637.666 | <0.0001 | -0.012 | -1469.406 |
| PWQ | <0.0001 | -0.250 | 0.782 | -1675.964 | <0.0001 | -0.097 | -1532.239 |
| Integrative climatic index |  |  |  |  |  |  |  |
| AET | <0.0001 | -0.368 | 0.735 | -1712.763 | <0.0001 | -0.148 | -1572.487 |
| Im | <0.0001 | -0.058 | 0.843 | -1644.748 | <0.0001 | -0.032 | -1483.471 |
| NPP | <0.0001 | -0.179 | 0.807 | -1660.857 | <0.0001 | -0.068 | -1509.869 |

4.2 Fiber fraction

| Predictor | Phylogenetic model | | | | Non-phylogenetic model | | |
| --- | --- | --- | --- | --- | --- | --- | --- |
|  | p-value | R_phy_^2^ | lambda | AIC | p-value | R^2^ | AIC |
| **Geography gradient** |  |  |  |  |  |  |  |
| Maximum longitude(MALO) | ***0.153*** |  |  |  | 0.089 |  |  |
| Minimum longitude(MILO) | ***0.033*** |  |  |  | 0.104 |  |  |
| Midpoint longitude(MPLO) | ***0.635*** |  |  |  | 0.921 |  |  |
| Maximum latitude(MALA) | <0.0001 | -0.102 | 0.710 | -2388.851 | <0.0001 | -0.041 | -2243.839 |
| Minimum latitude(MILA) | <0.01 | -0.072 | 0.711 | -2368.120 | <0.0001 | -0.041 | -2267.546 |
| Midpoint latitude(MPLA) | <0.0001 | -0.110 | 0.703 | -2385.798 | <0.0001 | -0.037 | -2279.402 |
|  |  |  |  |  |  |  |  |
| **Climate** |  |  |  |  |  |  |  |
| Thermal index |  |  |  |  |  |  |  |
| MAT | <0.0001 | 0.103 | 0.704 | -2381.365 | <0.0001 | 0.035 | -2239.842 |
| MTCM | <0.0001 | 0.105 | 0.701 | -2378.826 | <0.0001 | 0.034 | -2238.762 |
| MTWM | <0.0001 | 0.047 | 0.725 | -2370.759 | <0.0001 | 0.021 | -2229.891 |
| ABT | <0.0001 | 0.098 | 0.707 | -2382.245 | <0.0001 | 0.033 | -2238.528 |
| PET | <0.0001 | 0.081 | 0.713 | -2378.262 | <0.0001 | 0.025 | -2232.885 |
| WI | <0.0001 | 0.092 | 0.709 | -2380.876 | <0.0001 | 0.030 | -2236.219 |
| CI | <0.0001 | 0.080 | 0.708 | -2369.861 | <0.0001 | 0.032 | -2237.481 |
| Humid/arid index |  |  |  |  |  |  |  |
| MAP | <0.0001 | 0.081 | 0.718 | -2386.041 | <0.0001 | 0.042 | -2245.137 |
| PCQ | <0.05 | ***0.003*** | 0.740 | -2364.885 | <0.01 | 0.013 | -2223.752 |
| PWQ | <0.0001 | 0.061 | 0.721 | -2374.935 | <0.0001 | 0.027 | -2233.685 |
| Integrative climatic index |  |  |  |  |  |  |  |
| AET | <0.0001 | 0.104 | 0.709 | -2389.015 | <0.0001 | 0.042 | -2244.942 |
| Im | <0.05 | ***0.000*** | 0.752 | -2363.943 | <0.01 | 0.012 | -2223.034 |
| NPP | <0.05 | 0.046 | 0.721 | -2364.068 | <0.01 | 0.011 | -2222.873 |

4.3 Ray fraction

| Predictor | Phylogenetic model | | | | Non-phylogenetic model | | |
| --- | --- | --- | --- | --- | --- | --- | --- |
|  | p-value | R_phy_^2^ | lambda | AIC | p-value | R^2^ | AIC |
| **Geography gradient** |  |  |  |  |  |  |  |
| Maximum longitude(MALO) | <0.0001 | -0.049 | 0.671 | 2042.503 | <0.0001 | -0.032 | -1928.304 |
| Minimum longitude(MILO) | ***0.322*** |  |  |  | 0.081 |  |  |
| Midpoint longitude(MPLO) | <0.05 | ***-0.001*** | 0.685 | -2028.110 | <0.05 | -0.007 | -1910.228 |
| Maximum latitude(MALA) | <0.0001 | 0.116 | 0.643 | -2056.803 | <0.0001 | 0.046 | -1938.617 |
| Minimum latitude(MILA) | <0.05 | 0.062 | 0.653 | -2028.744 | <0.0001 | 0.039 | -1949.913 |
| Midpoint latitude(MPLA) | <0.0001 | -0.117 | 0.637 | -2049.941 | <0.0001 | -0.037 | -1967.058 |
|  |  |  |  |  |  |  |  |
| **Climate** |  |  |  |  |  |  |  |
| Thermal index |  |  |  |  |  |  |  |
| MAT | <0.0001 | 0.113 | 0.637 | -2046.361 | <0.0001 | 0.034 | -1929.457 |
| MTCM | <0.0001 | 0.119 | 0.638 | -2052.279 | <0.0001 | 0.041 | -1934.313 |
| MTWM | ***0.398*** |  |  |  | 0.172 |  |  |
| ABT | <0.0001 | 0.114 | 0.636 | -2046.238 | <0.0001 | 0.035 | -1930.301 |
| PET | <0.0001 | 0.106 | 0.637 | -2041.52 | <0.0001 | 0.033 | -1928.66 |
| WI | <0.0001 | 0.111 | 0.637 | -2045.225 | <0.0001 | 0.035 | -1930.093 |
| CI | <0.0001 | 0.068 | 0.655 | -2035.400 | <0.001 | 0.016 | -1916.753 |
| Humid/arid index |  |  |  |  |  |  |  |
| MAP | <0.0001 | 0.081 | 0.644 | -2030.850 | <0.001 | 0.016 | -1916.893 |
| PCQ | ***0.383*** |  |  |  | 0.610 |  |  |
| PWQ | <0.0001 | 0.078 | 0.646 | -2030.950 | <0.001 | 0.018 | -1917.982 |
| Integrative climatic index |  |  |  |  |  |  |  |
| AET | <0.0001 | 0.118 | 0.628 | -2038.481 | <0.0001 | 0.029 | -1926.147 |
| Im | ***0.882*** |  |  |  | 0.893 |  |  |
| NPP | <0.0001 | 0.072 | 0.658 | -2042.614 | <0.0001 | 0.032 | -1928.222 |

4.4 Axial parenchyma fraction

| Predictor | Phylogenetic model | | | Non-phylogenetic model | | |
| --- | --- | --- | --- | --- | --- | --- |
|  | p-value | R_phy_^2^ | AIC | p-value | R^2^ | AIC |
| **Geography gradient** |  |  |  |  |  |  |
| Maximum longitude(MALO) | ***0.693*** |  |  | 0.109 |  |  |
| Minimum longitude(MILO) | ***0.190*** |  |  | 0.704 |  |  |
| Midpoint longitude(MPLO) | ***0.254*** |  |  | 0.376 |  |  |
| Maximum latitude(MALA) | ***0.223*** |  |  | 0.072 |  |  |
| Minimum latitude(MILA) | ***0.987*** |  |  | 0.086 |  |  |
| Midpoint latitude(MPLA) | ***0.718*** |  |  | 0.053 |  |  |
|  |  |  |  |  |  |  |
| **Climate** |  |  |  |  |  |  |
| **Thermal index** |  |  |  |  |  |  |
| MAT | ***0.164*** |  |  | <0.05 | 0.007 | -1547.469 |
| MTCM | ***0.284*** |  |  | <0.05 | 0.007 | -1547.872 |
| MTWM | ***0.170*** |  |  | 0.283 |  |  |
| ABT | ***0.094*** |  |  | <0.05 | 0.006 | -1546.791 |
| PET | ***0.110*** |  |  | 0.099 |  |  |
| WI | ***0.087*** |  |  | 0.054 |  |  |
| CI | ***0.872*** |  |  | <0.05 | 0.007 | -1547.792 |
| **Humid/arid index** |  |  |  |  |  |  |
| MAP | ***0.224*** |  |  | 0.114 |  |  |
| PCQ | ***0.789*** |  |  | 0.769 |  |  |
| PWQ | ***0.348*** |  |  | <0.05 | 0.007 | -1547.743 |
| **Integrative climatic index** |  |  |  |  |  |  |
| AET | ***0.117*** |  |  | 0.103 |  |  |
| Im | ***0.338*** |  |  | <0.05 | 0.008 | -1548.660 |
| NPP | ***0.128*** |  |  | 0.376 |  |  |

4.5 Total parenchyma fraction

| Predictor | Phylogenetic model | | | | Non-phylogenetic model | | |
| --- | --- | --- | --- | --- | --- | --- | --- |
|  | p-value | R_phy_^2^ | lambda | AIC | p-value | R^2^ | AIC |
| **Geography gradient** |  |  |  |  |  |  |  |
| Maximum longitude(MALO) | <0.0001 | 0.049 | 0.758 | -2046.393 | <0.0001 | 0.037 | -1958.32 |
| Minimum longitude(MILO) | ***0.630*** |  |  |  | <0.05 | 0.006 | -1935.506 |
| Midpoint longitude(MPLO) | <0.01 | ***0.000*** | 0.773 | -2033.579 | <0.05 | 0.008 | -1936.935 |
| Maximum latitude(MALA) | <0.0001 | 0.132 | 0.724 | -2054.046 | <0.0001 | 0.056 | -1971.655 |
| Minimum latitude(MILA) | <0.05 | 0.061 | 0.744 | -2032.135 | <0.001 | 0.021 | -1946.233 |
| Midpoint latitude(MPLA) | <0.0001 | 0.128 | 0.723 | -2049.313 | <0.0001 | 0.050 | -1967.32 |
|  |  |  |  |  |  |  |  |
| **Climate** |  |  |  |  |  |  |  |
| Thermal index |  |  |  |  |  |  |  |
| MAT | <0.0001 | 0.124 | 0.758 | -2043.469 | <0.0001 | 0.044 | -1962.993 |
| MTCM | <0.0001 | 0.132 | 0.721 | -2049.847 | <0.0001 | 0.052 | -1968.85 |
| MTWM | ***0.850*** |  |  |  | 0.073 |  |  |
| ABT | <0.0001 | 0.124 | 0.720 | -2042.207 | <0.0001 | 0.043 | -1962.629 |
| PET | <0.0001 | 0.106 | 0.726 | -2038.006 | <0.0001 | 0.038 | -1958.918 |
| WI | <0.0001 | 0.120 | 0.722 | -2041.063 | <0.0001 | 0.042 | -1961.923 |
| CI | <0.0001 | 0.075 | 0.742 | -2038.780 | <0.0001 | 0.027 | -1950.549 |
| Humid/arid index |  |  |  |  |  |  |  |
| MAP | <0.05 | 0.089 | 0.731 | -2031.883 | <0.0001 | 0.022 | -1947.191 |
| PCQ | ***0.379*** |  |  |  | 0.740 |  |  |
| PWQ | <0.0001 | 0.099 | 0.727 | -2033.121 | <0.0001 | 0.028 | -1951.573 |
| Integrative climatic index |  |  |  |  |  |  |  |
| AET | <0.01 | 0.114 | 0.722 | -2036.312 | <0.0001 | 0.035 | -1956.708 |
| Im | ***0.236*** |  |  |  | <0.05 | 0.006 | -1935.803 |
| NPP | <0.0001 | 0.066 | 0.746 | -2038.473 | <0.0001 | 0.031 | -1953.542 |

**Appendix 5**

Summary of decomposition of covariance in a general linear model, using sums of products, for the effects of climate (MAT, MTCM and AET) and phylogenetic variation (1st-order grouping and 2nd-order grouping) on the five xylem tissue fractions (VF, BF,RF, AF and RAF). Explanatory terms are listed in the order of their entry into the model. Df: degree of freedom, %SP: percentage of total sum of products, Sig.: significance, ***: P ≤ 0.001, **: P ≤ 0.01, *: P ≤ 0.05, NS: P > 0.05.

|  | Df | %SP | Sig. | Df | %SP | Sig. | Df | %SP | Sig. | Df | %SP | Sig. | Df | %SP | Sig. |
| --- | --- | --- | --- | --- | --- | --- | --- | --- | --- | --- | --- | --- | --- | --- | --- |
|  | VF | | | FF | | | RF | | | AF | | | RAF | | |
| Climate first model | | | | | | | | | | | | | | | |
| MAT | 1 | 14.591 | *** | 1 | 3.507 | *** | 1 | 3.385 | *** | 1 | 0.653 | ** | 1 | 4.388 | *** |
| MTCM | 1 | 0.682 | ** |  |  |  |  |  |  |  |  |  | 1 | 1.278 | *** |
| AET | 1 | 1.149 | *** | 1 | 0.852 | ** | 1 | 1.124 | *** |  |  |  |  |  |  |
| 1st-order grouping | 84 | 30.526 | *** | 84 | 34.545 | *** | 84 | 28.378 | *** | 84 | 34.600 | *** | 84 | 22.470 | ** |
| 2nd-order grouping | 209 | 20.247 | NS | 209 | 24.643 | * | 209 | 26.786 | * | 209 | 37.584 | *** | 209 | 33.688 | *** |
| Residual | 403 | 32.805 |  | 404 | 36.452 |  | 404 | 40.328 |  | 405 | 27.164 |  | 404 | 38.177 |  |
| Phylogeny first model | | | | | | | | | | | | | | | |
| 1st-order grouping | 84 | 36.050 | *** | 84 | 34.988 | *** | 84 | 28.872 | *** | 84 | 35.157 | *** | 84 | 24.788 | *** |
| 2nd-order grouping | 209 | 25.860 | *** | 209 | 26.045 | ** | 209 | 29.255 | ** | 209 | 37.298 | *** | 209 | 35.401 | *** |
| MAT | 1 | 4.528 | *** | 1 | 1.425 | *** | 1 | 1.260 | *** | 1 | 0.381 | * | 1 | 1.068 | *** |
| MTCM | 1 | 0.056 | NS |  |  |  | 1 | 0.2860 | NS |  |  |  | 1 | 0.566 | * |
| AET | 1 | 0.702 | ** | 1 | 1.090 | *** |  |  |  |  |  |  |  |  |  |
| Residual | 403 | 32.804 |  | 404 | 36.452 |  | 404 | 40.328 |  | 405 | 27.164 |  | 404 | 38.177 |  |
